# Assessment of the Dimensional and Geometric Precision of Micro-Details Produced by Material Jetting

**DOI:** 10.3390/ma14081989

**Published:** 2021-04-15

**Authors:** Miguel R. Silva, António M. Pereira, Álvaro M. Sampaio, António J. Pontes

**Affiliations:** 1 Institute for Polymers and Composites-IPC, School of Engineering, University of Minho, 4800-058 Guimarães, Portugal or amsampaio@arquitetura.uminho.pt (Á.M.S.); pontes@dep.uminho.pt (A.J.P.); 2 CDRSP, ESTG, Polytechnic of Leiria, 2401-951 Leiria, Portugal; mario.pereira@ipleiria.pt; 3 Lab2PT, School of Architecture, University of Minho, 4800-058 Guimarães, Portugal

**Keywords:** additive manufacturing, precision, polyjet, polymers

## Abstract

Additive Manufacturing (AM) technology has been increasing its penetration not only for the production of prototypes and validation models, but also for final parts. This technology allows producing parts with almost no geometry restrictions, even on a micro-scale. However, the micro-Detail (mD) measurement of complex parts remains an open field of investigation. To be able to develop all the potential that this technology offers, it is necessary to quantify a process’s precision limitations, repeatability, and reproducibility. New design methodologies focus on optimization, designing microstructured parts with a complex material distribution. These methodologies are based on mathematical formulations, whose numerical models assume the model discretization through volumetric unitary elements (voxels) with explicit dimensions and geometries. The accuracy of these models in predicting the behavior of the pieces is influenced by the fidelity of the object’s physical reproduction. Despite that the Material Jetting (MJ) process makes it possible to produce complex parts, it is crucial to experimentally establish the minimum dimensional and geometric limits to produce parts with mDs. This work aims to support designers and engineers in selecting the most appropriate scale to produce parts discretized by hexahedral meshes (cubes). This study evaluated the dimensional and geometric precision of MJ equipment in the production of mDs (cubes) comparing the nominal design dimensions. A Sample Test (ST) with different sizes of mDs was modeled and produced. The dimensional and geometric precision of the mDs were quantified concerning the nominal value and the calculated deviations. From the tests performed, it was possible to conclude that: (i) more than 90% of all analyzed mDs exhibit three dimensions (xyz) higher than the nominal ones; (ii) for micro-details smaller than 423 μm, they show a distorted geometry, and below 212 μm, printing fails.

## 1. Introduction

AM technology makes it possible to produce highly complex components, layer-by-layer, with few geometric restrictions when compared with conventional manufacturing processes. With technological advances in processes and materials, AM has been adopted as a technology, not only for the production of prototypes (rapid prototyping), but also in the production of functional parts [1,2,3]. Among the advantages offered by AM, we can highlight: (i) the geometric freedom of parts without the need to use other tools; (ii) the reduced product development time [4]; (iii) the decrease in the cost associated with the parts’ complexity or their customization [5]; and (iv) unleashing the potential for components’ functionalization [6,7,8].

The effective adoption of AM processes requires, first, alongside the characterization of the produced parts’ properties, the knowledge of the processing capacity (i.e., limits). We can consider the process’s maximum resolution a lower limit to the production of mDs. From a practical point of view, the resolution indicated by the equipment manufacturers refers to the minimum amount of material that the process allows for manufacturing, without however indicating its geometry, which depends on the nature of the process (e.g., it is not a perfect cube or cuboid) [9,10]. On the other hand, the minimum amount of material to be deposited has a finite size, so the smaller the details to produce, the greater the deviations (higher or lower) are [11]. Another aspect to consider is the preprocessing errors that occur when converting a solid model into layer-by-layer file production (slicing), which inevitably are the result of approximations (e.g., .stl). The sum of these local errors (i.e., errors per layer) translates into global errors [11]. Thus, considering that these characteristics influence the production of parts with mDs, it is assumed to be relevant to quantify experimentally the geometric and dimensional deviations that occur in their production.

One of the oldest AM processes, but which remains current, is the MJ process (this process derives from inkjet printing invented in the 1950s for use in typewriters and later in the 1980s in ink-jet printers) [12]. This process is characterized by the ability to control the localized deposition of drops of a layer-by-layer material, with high resolution, dimensional, and geometric rigor [13]. Due to its characteristics, this process has been used in several application areas to manufacture products with advanced features, such as: (i) biomedical engineering [14,15,16,17]; (ii) wearables [18,19]; and (iii) electronics [20,21]. Several companies have developed MJ variants: The Polyjet™ process developed by Stratasys, USA, is one of those examples. The high resolution and precision of the Polyjet™ process, combined with the different materials available, allows the expansion of the application areas as in the case of micro-Additive Manufacturing (μAM) [9,22].

**Figure 1:**
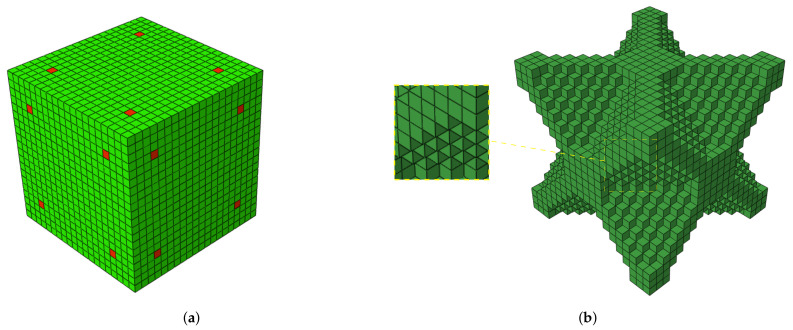
TO design domain discretization using an FEM hexahedral mesh (cubes): (**a**) initial design domain; (**b**) final optimized topology.

The design and product development for AM production adopt dedicated methodologies (i.e., specific), which allow, on the one hand, unlocking the potential of the technology (i.e., different processes) and, on the other, ensuring the rigor between the model and the final product. From the perspective of design engineering, the ability to produce internal microstructured parts extends the design space. In recent years, several studies have explored the complex micro-mechanical behavior (i.e., new properties) that result from the spatial material distribution inside the part. Recent work explored the design of new digital materials represented by voxels [23,24] using computational tools, such as Topological Optimization (TO) algorithms. Several methods for TO have been developed, namely the homogenization method [25,26], the Evolutionary Structural Optimization (ESO) method [27,28], Bidirectional ESO (BESO) [29,30], Genetic Algorithm (GA) [31,32], Level Set Methods (LSMs) [33,34], and Solid Isotropic Material with Penalization (SIMP) [35]. Generally, these TO methods are based on the numerical Finite Element Method (FEM) to solve the TO problem, typically by discretizing the design domain into a large number of finite elements, e.g., hexahedral (Figure 1).

In these microstructured materials, the mechanical properties are dependent on the spatial distribution inside the material, allowing the production of parts with superior performance [36] and multifunctional responses [37,38]. The AM production of microstructured parts (i.e., voxels) requires, on the one hand, the precise distribution of the material within the microstructure and, on the other hand, that the voxels have dimensional and geometric rigor with respect to the model. Despite being possible to theoretically determine the minimum voxel size for the different AM processes, these calculations in general do not consider their real complexity. Variations in equipment components, material properties, quality, and external disturbances (e.g., vibrations, variations in temperature, and humidity) can influence the performance of systems [39]. On the other hand, the nature of the process imposes a specific geometry to the minimum voxel. In the case of the Polyjet™ process, when we approach the lower resolution limit, filling a surface with circumferences (i.e., drop shape) will cause an error (i.e., by excess or defect), and this error will decrease with the scale increase of the smallest part detail. The true process precision and resolution cannot be determined without experimentally producing, measuring, and characterizing the parts with dimensions details close to the system’s minimum voxel size.

This work presents a study of the dimensional and geometric error of an ST with mDs, cubes produced by the Polyjet™ process. Next, the process is described, and a review of the existing STs’ bibliography and process application studies in μAM is given. The Materials and Methods Section describes the equipment, pre-processing, production, and post-processing procedures. The ST is described, which is the methodology for measuring and calculating deviations. The fourth section presents the results and discusses them. Finally, conclusions are presented.

### Background

The AM process MJ consists of the direct deposition of a jet of photo polymeric acrylic resin (monomers, oligomers, and photoinitiator) and later curing by UV light. The machine Connex Objet500™ (Stratasys, Minneapolis, MN, USA) has eight printheads (i.e., eight parallel lines with 100 nozzles each) and can simultaneously print three materials: four heads dedicated to the support material, the other four allowing the use of two different construction materials. A roller on the printing block levels the resin deposited on the layer to provide a flat surface for the deposition of the subsequent layer. To provide support and stability for resin droplets, printed parts can be completely involved by the support material. The top surfaces are also, by default, coated in support material to provide a uniform surface finish (i.e., matte); with this option not selected, the upper surface remains as a glossy finish. The support material is composed of a mixture of polypropylene, polyethylene, and glycerin. It can be removed mechanically, by high-pressure water jet, or by dissolving in a chemical bath (2% NaOH), (i.e., Material Refs. SUP705 and SUP706, respectively). The Connex Objet500™ has a 42 μm (600 dpi) resolution in the build platform plan (XY) and a 32 μm or 16 μm layer height in “High Speed mode” or “High Quality mode”, respectively [40]. Table 1 shows a summary of the Connex3 Objet500™ specifications.

The Polyjet™process has been used in several application, in the production of prototypes and functional polymeric parts, such as: (i) honeycomb components; (ii) customized anatomical models; (iii) scaffolds; and (iv) wearables [8]. Despite the high theoretical resolution, in practice, the part mDs cannot be produced with this resolution due to the nature of the printing process (e.g., droplet size and shape, recoater action). The parts produced using the Polyjet™ technology have low anisotropy (≈2%) when compared with other AM processes. This behavior is due to the fact that the local volume is compacted by the deposited liquid resin, as well as by the process using a low amount of curing energy, promoting uniform polymerization of the entire volume [41]. Despite this fact, in reality, the resolution is less than theoretically defined. To guarantee the fidelity (i.e., geometries, dimensions, and properties) between models and final products, it is necessary to develop knowledge of the process, materials, and produced parts’ characteristics.

Several studies have been presented regarding the process characterization [42,43,44,45], the influence of processing parameters [46,47,48,49,50], and the thermomechanical properties [51,52,53]. The part quality analysis (i.e., dimensional and geometric accuracy of the mDs) is another important area to establish the design rules. Several authors investigated the quality of the parts produced by AM, quantifying the surface quality and the mDs’ dimensional accuracy. Several other researchers have proposed the use of STs to benchmark quantitatively AM processes’ capacities. Cooke et al. [54] used ST NAS 979 (Sample Test developed in 1966 by the Aerospace Industries Association– AIA) to quantify the geometric errors of the pieces produced by AM. As this test was originally designed for conventional milling parts, this study did not manage to quantify the parts mDs produced by AM. More recently, Moylan et al. [55] proposed an ST, which intends to incorporate most of the geometric shapes and dimensions required to test the capacity of different AM processes and equipment. Some comparative studies use STs with geometries and details intended to measure the dimensional and geometric precision of the parts produced by AM [56,57,58,59] or the equipment and process performance in relation to the production speed and efficiency [58]. Other works establish the correlation between nominal geometry and dimensions (i.e., design) with the corresponding real parts’ dimensional and geometric tolerance [60,61,62,63,64]. Nevertheless, dimensional and geometric tolerance in AM still presents challenges derived from specific aspects such as: (i) manufacturing direction; (ii) localization in the build envelope; (iii) layer thickness; and (iv) support structures, in the tolerance of complex surfaces, topologically optimized structures, and internal details [65]. In addition, certain applications require specific STs. An example is the microfluidics research field (i.e., chip production) for which STs were developed to assess the resolution, precision, and repeatability of MJ equipment [17,66,67,68,69].

**Figure 2:**
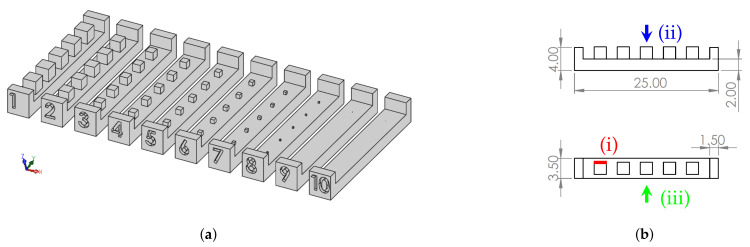
Sample test specimens’ CAD model and 2D drawings with dimensions in μm: (**a**) solid model (trimetric view); (**b**) STs’ 2D drawings with cube edge (**i**), top view (**ii**), and side view (**iii**) highlighted.

This work presents a study of the dimensional and geometric error of cube-shaped mDs, produced by the Polyjet™ process. To quantify the accuracy of the equipment, ten STs with different mDs were modeled according to Figure 2a. Each ST had five same-sized cubes, evenly distributed under the base surface. STs were produced by the Polyjet™ process in VeroClear material (Stratasys, Minneapolis, MN, USA) with and without support material. After, they were observed under a microscope and proceeded to the acquisition of enlarged images. Subsequently, the dimensions and geometric shapes were analyzed using image analysis software [70]. Lastly, the error was calculated and compared to the nominal dimensions. In the following section, the materials and methods used in this work are presented.

## 2. Materials and Methods

In this study, ten STs were considered, and each of the STs contained five same-sized mDs (cubes) equally spaced on a base of dimensions 25×3.5×2 mm${}^{3}$ (Figure 2b).

For MD (i.e., cube edge), the dimensions’ selection was considered a multiple of the equipment minimum resolution (42.33 μm). This set consisted of ten STs (S01 to S10) with mD dimensions (i.e., cube edge) ranging from 2117 μm to 21 μm. The specimens S01 to S07 were produced without support material. For the specimens S01S to S07S produced with support material, the suffix “S” was added to the reference name (SXXS) (Table 2).

**Figure 3:**
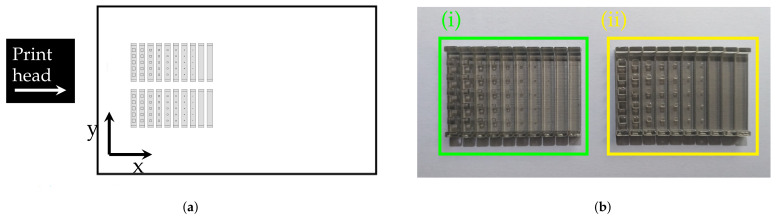
Polyjet tray showing the STs’ positing; (**a**) (Ph) Polyjet build orientation (i.e., printhead movement direction); (**b**) STs after support removal with highlight selection considered for analysis; (**i,ii**) specimens produced without and with support material, respectively.

STs were modeled using the CAD software SolidWorks 2019 (Dassault Systemes SolidWorks Corporation) (Figure 2a). The files were converted to the .stl format, with a conversion tolerance of 1.0 μm, and manufactured in a Stratasys Connex3 Objet500™. STs were produced with VeroClear RGD810™ thermosetting polymer and the base supports in SUP705, with a 16 μm layer height resolution. Production was carried out under the following conditions: (i) STs positioned on the xy plane, with the largest dimension along the yy axis (Figure 3a); (ii) surface finishing “gloss mode” (no supporting material to wrap the part) and “matte mode”; (iii) resins were stored in a controlled environment, prior to being placed in the equipment, according to the supplier rules; (iv) after production, support material was removed in a chemical bath of 2% sodium hydroxide (NaOH) and 1% sodium metasilicate (Na${}_{2}$SIO${}_{3}$) at room temperature for 5 h.

### Optical Image Acquisition and Analysis

**Figure 4:**
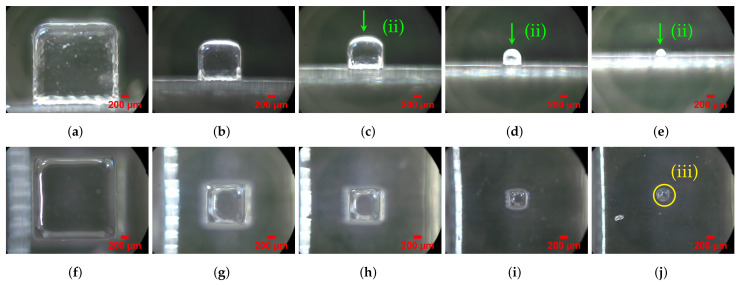
mD specimens S01, S04, S05, S07, and S08 (produced without support material). (**ii**) The radius corner influence in geometry deviations increases for small features. For dimensions smaller than S04 1058 μm, (**b**) the top side became domed; (**iii**) for the size S08, the cube was distorted in cylindrical shape; (**a**,**f**) S01 side and top view; (**b**,**g**) S04 side and top view; (**c**,**h**) S05 side and top view; (**d**,**i**) S07 side and top view; (**e**,**j**) S08 side and top view. For details about the conditions of the images taken, see Section 2.

ST images (50× magnification) were obtained through an optical microscope Zeiss AxioTech 100HD (Zeiss, Oberkochen, Germany) . A CanonCamPS camera was used with 3648×2736 pixel resolution, and the files were saved in the .jpeg extension. Two images were taken, i.e., vertical and lateral (Figure 4j), for each mD selection considered for analysis (Figure 3b).

As a criterion, details that presented orthogonal adjacent sides were considered valid for analysis, despite the rounded vertices. Three mD dimensions were analyzed according to 3 axes, X (XDim), Y (YDim), and Z (ZDim), and the projected area, in two planes, the xy horizontal plane (AreaH) and the yz vertical plane (AreaV), respectively (Figure 3a). The mD measurements were made with the image analysis software ImageJ [70]. For XDim, YDim, and ZDim, five measurements were made in each of the five details of the same size, for a total of 25 measurements (5 cubes × 5 measurements) per size. The areas dimensions AreaH and AreaV were measured once in detail, for a total of 5 measurements per size (5 cubes × 1 area). Based on these data, a statistical analysis was performed, and the mean and Standard Deviation (*SD*) values were calculated. The Relative error percentage (Re %) was calculated based on the measure regarding the nominal dimensions (Re % $=\frac{measured\;value-nominal\;value}{nominal\;value}\times 100$). The geometric assessment was performed based on ST observation (Figure 4j). A heuristic methodology was also applied to quantify the geometric deviations resulting from the rounded vertices and burr edges. The Geometric Heuristics (GHeuH and GHeuV) assessed the relative error between the measured area and that obtained by multiplying the projected cube XDim × YDim, and YDim × ZDim for the horizontal and vertical plane, respectively.

## 3. Results and Discussion

The results were obtained by comparing the dimensions and geometries of the details produced with the nominal values of the CAD model. In Figure 3b is the STs’ enlarged image, with emphasis on the numbered selection of details considered valid according to established criteria.

For this study, only the first seven STs with dimensions (i.e., edge) between 2117 μm and 423 μm were considered. For the STs produced, it was observed that of the 10 STs, the seventh (423 μm) and eighth (212 μm) were the last (i.e., smaller dimensions) to be fully produced with and without support material, respectively (Figure 3b).

### 3.1. Specimens Produced without Support Material

For specimens produced without support material, the eighth size (S08) inclusive cube (212 μm) was distorted and took on a cylindrical shape in the horizontal projection (top view), assuming a domed top surface in the vertical projection (side view). This effect may result from the inability of the equipment to produce vertices without rounding (i.e., geometry sharpness of edges) (Figure 4).

**Figure 5:**
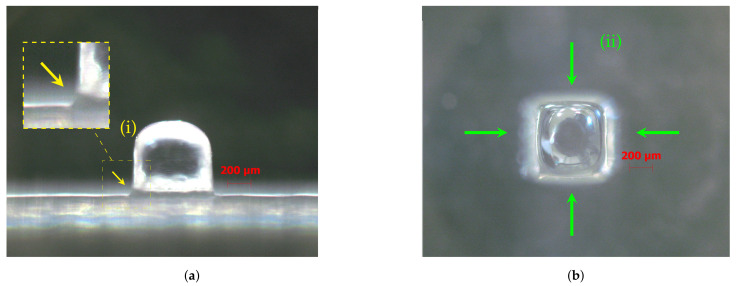
Specimen S06 detail. (**a**) Side view, (**i**) excess material in the specimen base; (**b**) top view, (**ii**) outside double perimeter outline. For details about the conditions of the images taken, see Section 2.

All details had a double perimeter outline (Figure 5), apparently indicating that the dimensions of the top of the cube were smaller than those of the base (i.e., there was a passing deviation on the edge between the cube and the base). This effect was described by [8], which attributed the cause to the possible spreading of the resin before being polymerized.

**Figure 6:**
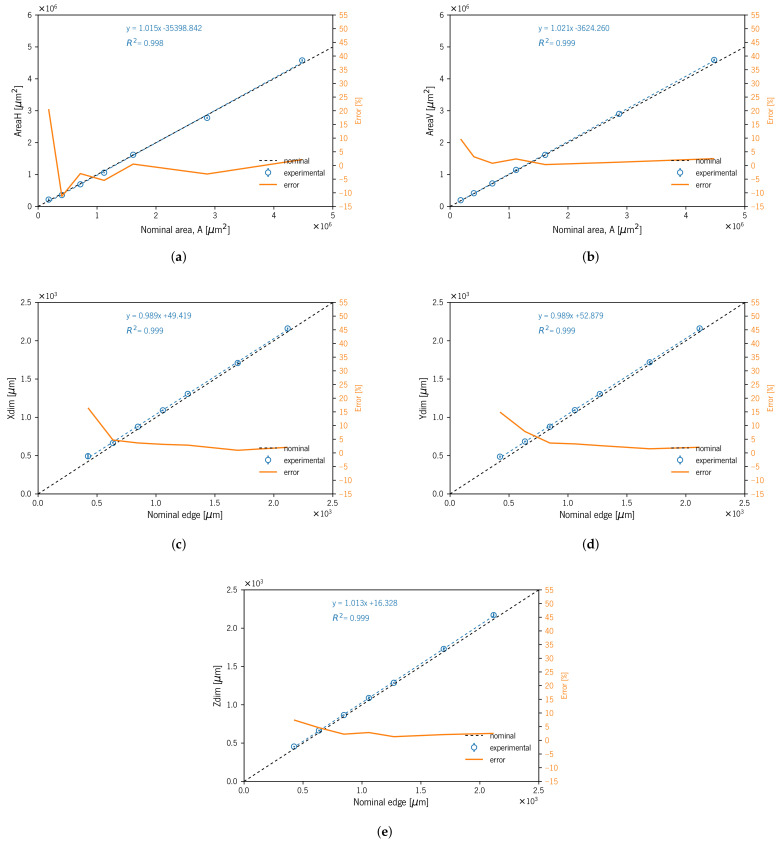
Experimental mDs’ measurement diagrams with linear fit and relative error for specimens A01 to A07: (**a**) AreaH; (**b**) AreaV; (**c**) XDim; (**d**) YDim; (**e**) ZDim.

Table 3 presents the average results (± standard deviation) regarding the five measured experimental dimensions. The measured mDs’ characteristic dimensions XDim, YDim, and ZDim were greater than the nominal dimensions (Table 3 and Figure 6). The S01 to S05 XDim Re % varied between 0.96 and 3.63% for S02 and S05, respectively. The S06 and S07 specimens presented 4.7 and 16.7% Re %, respectively. Considering all three dimensions XDim, YDim, and ZDim, the Re % varied between ≈ 1–3%. However, for small dimensions (S06 and S07), the deviation increased. The specimen S07 exhibited the maximum Re % of 16.47, 14.92, and 7.44% for XDim, YDim, and ZDim, respectively. The maximum *SD* was 40 μm for size S07 (423 μm) on XDim, and it should be noted that all the other values corresponded to less than half the nominal value of the 600 dpi resolution (42.33 μm) indicated by the equipment manufacturer (Table 1).

The results for the AreaH dimension showed Re% deviations between −11.28 and 20.61% for sizes S06 and S07, respectively. The data for this dimension were obtained through a dedicated measurement. However, as mentioned before, the horizontal projected image showed an outside double perimeter outline due to this area measurement being prone to error. This fact showed a greater influence on smaller specimens: for sizes between S01 and S05, the Re% varied from −5.45 to 2.16%, respectively. On the contrary, AreaV showed a smaller Re% deviation; if considering only S01 to S06, the Re% range was 0.8 to 3.18% for S05 and S06, respectively. In this dimension, the Re% showed smaller values and variation than those observed in the previous dimension (i.e., AreaH).

**Figure 7:**
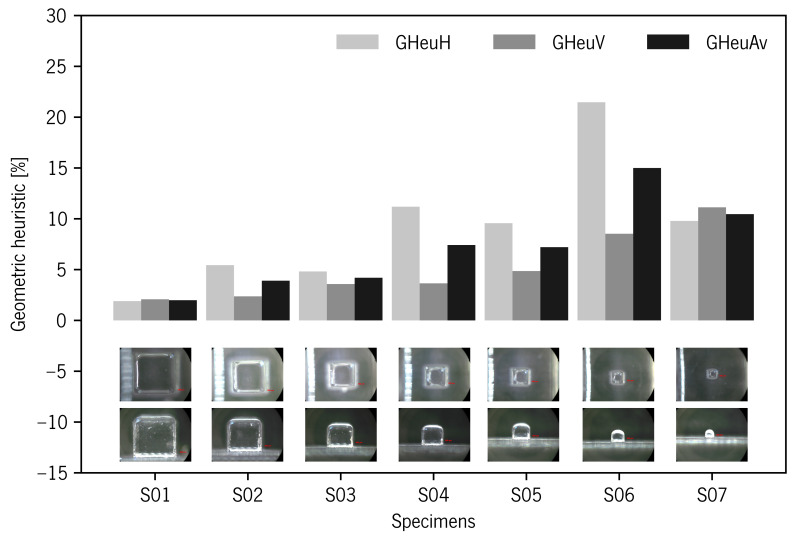
Geometric heuristic diagram for specimens produced without support material. Below the diagram, the representative images corresponding to each specimen.

The geometry visual assessment showed some slight rounding of sharp edges and corners on top surfaces. This passing deviation took on greater relevance with the specimen size decrease (Figure 4). The geometric heuristics GHeuH and GHeuV values showed consistency with project area reduction that occurred in the edges due to undercutting deviation (i.e., rounded edges). GHeuV showed that Re% deviations increased with decreasing specimen size, from 2.08 to 11.13% for specimens S01 and S07, respectively (Table 3 and Figure 7).

### 3.2. Specimens Produced with Support Material

**Figure 8:**
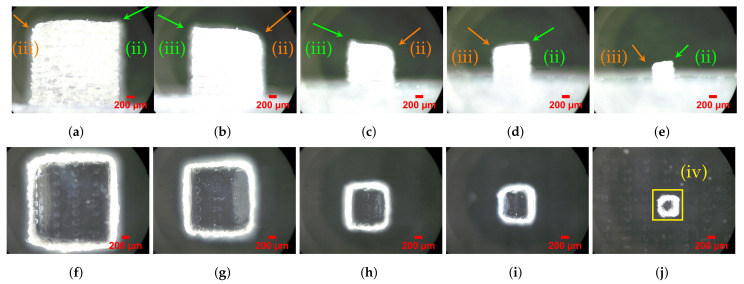
mD specimens A01S to A07S (produced with support material). The side walls exhibit a non-uniform surface finish (granulated satin). The top surface shows random geometrical deviations, similar to burr edges (**ii**) and rounded corners (**iii**), outside the ideal geometrical shape. (**iv**) For the size S07, the cube shows some geometrical distortion from a cube shape. (**a**,**f**) S01S side and top view; (**b**,**g**) S02S side and top view; (**c**,**h**) S04S side and top view; (**d**,**i**) S05S side and top view; (**e**,**j**) S07S side and top view. For details about the conditions of the images taken, see Section 2.

**Figure 9:**
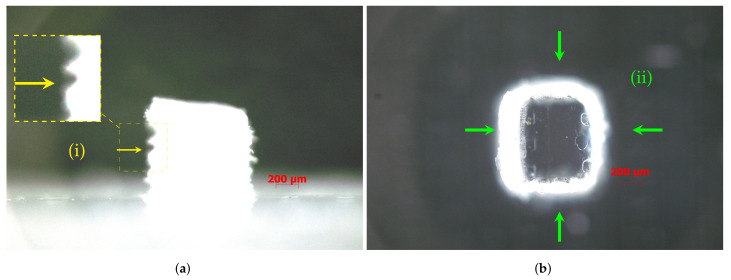
Specimen (S05S) detail: (**a**) side view (**i**) showing the side walls’ roughness and non-uniform surface finish; (**b**) top view (**ii**) showing over-thickness side walls and geometrical distortion. For details about the conditions of the images taken, see Section 2.

The use of support material theoretically allows the production of parts with greater geometrical and dimensional accuracy. However, the contact between two different materials and the nature of the Polyjet™ process (i.e., model and support material droplet spreading and recoater) will translate into an alteration, especially for part’s outer surfaces (interface). Specimens produced with support material compared to those previously described (i.e., produced without supports) showed similar shape distortions for S07S in the horizontal projection. However, in the vertical projection, the top surface showed significant changes in its profile. The vertices displayed random geometric deviations similar to blur and rounded corners (Figure 8), and these effects may be due to the interaction between the material model and support. Figure 9a shows the sidewalls’ roughness; this effect is more noticeable with the decrease of the part size and may lead to the weakness of the part. This surface roughness of the specimens produced with support material is inherent to the Polyjet™ process, as reported in another study [71].

Table 3 presents the measured average mD characteristics’ dimensions XDim, YDim, and ZDim. All three dimensions presented higher values than nominal. The five bigger XDim mDs’ (S01S–S05S) Re% varied between 7.5 and 13.96% for S04S and S01S, respectively. These differences between experimental and nominal dimensions were reported in another study [8]. The smaller mD S06S and S07S specimens presented Re%−0.71 and 13.59%, respectively. For YDim and ZDim, the Re% varied between 2.61 and 6.38% if excluding S05S, S06S, and S07S. YDim and ZDim showed a smaller overall Re% compared with XDim; this may be related to the fact that XDim coincides with the recoater movement direction, and this contact may contribute to dragging the material in this direction, as mentioned in another study [8]. S07S exhibited the maximum Re% of 23.59, 23.39, and 22.17% for XDim, YDim, and ZDim, respectively. The *SD* for the XDim, YDim, and ZDim measurements presented higher values compared to specimens produced without support material. The maximum *SD* for XDim, YDim, and ZDim was 57 μm for size S07S on XDim; it should be noted that in fifteen out of twenty-one, the values corresponded to less than the nominal value of the 600 dpi resolution (42.33 μm) indicated by the equipment manufacturer (Table 1).

The AreaH dimension results showed deviations from the nominal value between −1.46 and 45.23% for sizes S06S and S07S, respectively. The data for this dimension were obtained through a dedicated measurement. However, as mentioned before, the horizontal projected image showed an over-thickness wall (i.e., XDim and YDim measurements) along with some amount of geometrical distortion; due to these facts, the area measurement was prone to higher deviations. This fact showed a greater influence on bigger specimens: for sizes between S01S and S05S, the Re% varied from 18.38 to −1.46%, respectively. The AreaV dimension showed the Re% ranging between 6.59 and 18.41% for S01S and S05S, respectively, excluding S07S, which exhibited 49.22%, a similar value to the previous AreaV. These deviations could be related to some amount of geometrical distortion in the corners and edges similar to burr deviations (Figure 8 and Figure 9).

**Figure 10:**
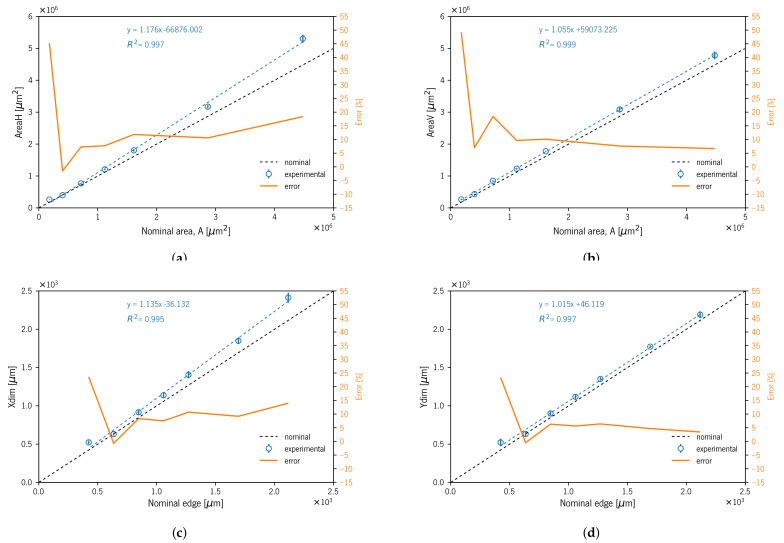

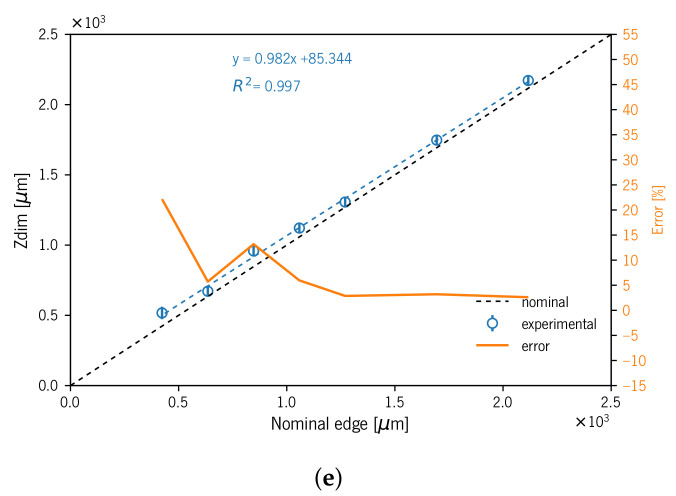
Experimental mD measurements diagrams with linear fit and relative error for specimens A01S to A07S: (**a**) AreaH; (**b**) AreaV; (**c**) XDim; (**d**) YDim; (**e**) ZDim.

**Figure 11:**
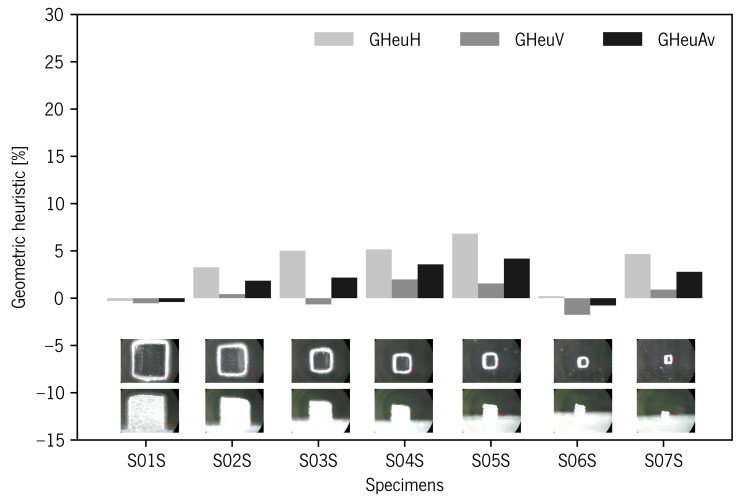
Geometric heuristic diagram for specimens produced with support material. Below the diagram, the representative images corresponding to each specimen.

The geometry visual assessment showed random rounded corners and burr edges on top surfaces. This deviation took on greater relevance with the specimen size decrease (Figure 10j). In Table 3 and Figure 11, the geometric heuristics GHeuH and GHeuV values showed consistency with fluctuations in the projected area values occurring in the mDs’ outer surfaces and edges due to burr and rounded corner deviations. GHeuH showed deviations from −0.29 to 6.82% for specimens S01S and S06S, respectively. GHeuV showed relative errors from −1.76 to 1.99% for S06S and S04S, respectively.

**Figure 12:**
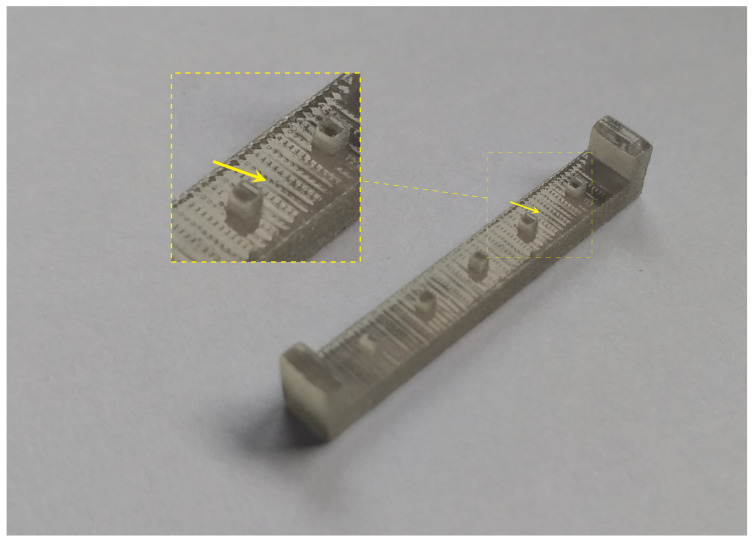
ST detail with highlighted marks/grooves on the top surface.

Figure 12 shows visible marks/grooves on the ST top surface. This effect occurred in both specimen groups (i.e., with and without support material). This consequence could be caused by the leveler (i.e., recoater) movement.

Figure 6 and Figure 10 show the linear correlations for all the specimens produced (with and without support material) between the measured XDim, YDim, ZDim, AreaH, and AreaV and the corresponding nominal dimensions with high coefficients of determination ($R^{2}$). Furthermore, we show the linear correlation between the five measured dimensions of mDs and the CAD model (nominal dimensions).

## 4. Conclusions

In this study, an ST with different sizes of mDs was produced in VeroClear™ using Polyjet™ technology. Optical equipment (i.e., microscope) was used to enlarge and acquire the image, and subsequently, an image analysis software was used to quantify the dimensions: XDim, YDim, ZDim, AreaH, and AreaV (A) of mDs of different sizes. The Standard Deviation (*SD*) and Relative error percentage (Re%) for nominal dimensions were also calculated. The smallest mD produced, according to the established criteria, measured 423 μm along the nominal edge. The results showed that more than 90% of the XDim, YDim, and ZDim analyzed had higher values than the nominal ones. Specimens produced without support material showed better precision.

For specimens produced without support material, a 212 μm edge cube was distorted assuming a cylindrical shape with a domed top surface in vertical projection. This may result from the equipment’s inability to produce vertices without rounding. For dimensions smaller than 212 μm, the equipment failed to produce the mDs. The advantages of printing without support material are smooth surface finishing, cost reduction (support material), and time savings in support removal.

Specimens produced with support material showed a similar shape distortion for size S07S (423 μm) in the horizontal projection. However, in vertical view, the top surface showed vertices with random geometric deviations similar to burr and rounded corners. The outer surfaces exhibited non-uniform surface finishing. The use of support material theoretically allows the production of parts with greater geometrical and dimensional accuracy. However, the contact of two different materials (model and support) and the nature of the PolyJet process (i.e., material droplet spreading and recoater) will translate into alteration, especially in the part outer surfaces (interface). In larger pieces, the impact of these changes will be in the surface quality (roughness, smooth surface). However, in smaller pieces, it can compromise its performance, and this effect is more significant with the reduction of the size of the pieces and with the increase of the ratio between the outer surface and volume.

Linear correlations between the measured XDim, YDim, ZDim, AreaH, and AreaV and the corresponding nominal dimensions with high coefficients of determination ($R^{2}$) allowed us to predict and estimate the real dimensions of the mDs. These data can be used as a design guideline to produce more accurate complex small detail parts, taking into account the possible deviations that occur during the manufacturing process.

## Figures and Tables

**Table 1:** Connex3 Objet500™ specifications [40].

Parameter	Value
Build envelope (mm)	490×390×200
Resolution XYZ (dpi)	600×600×1600
Resolution XYZ (μm)	42×42×16
Precision (μm)	20–200
Minimum layer height (μm)	16, 32
Minimum wall (mm)	0.6
Production Mode (μm)	DM: 32
	HQ: 16
	HS: 32

Note: DM, Digital Material; HQ, High Quality; HS, High Speed.

**Table 2:** Specimens’ nomenclature and dimensions.

	Specimen									
	S01	S02	S03	S04	S05	S06	S07	S08	S09	S10
Edge (μm)	2117	1693	1270	1058	847	635	423	212	42	21

Note: All specimens were produced with and without support material.

**Table 3:** Comparison between the CAD model (nominal) and the produced cubes’ dimensions.

Specimen	Model CAD		Produced Structures Measurements					Geometric Heuristic (%)		
	Edge (μm)	A (μm${}^{2}$)	XDim (μm)	YDim (μm)	ZDim (μm)	AreaH (μm${}^{2}$)	AreaV (μm${}^{2}$)	GHeuH	GHeuV	GHeuAv
S01	2117	4,480,292	2160±12	2160±15	2171±24	4,576,959±35,667	4,591,893±37,956	1.90	2.08	1.99
S02	1693	2,867,366	1709±19	1719±14	1729±20	2,777,847±32,263	2,901,815±10,155	5.44	2.37	3.91
S03	1270	1,612,900	1306±10	1304±13	1287±11	1,620,993±27,133	1,618,097±14,243	4.82	3.58	4.20
S04	1058	1,120,062	1091±9	1093±11	1089±19	1,058,980±19,173	1,146,775±26,889	11.19	3.65	7.42
S05	847	716,850	877±8	877±19	866±9	695,550±8792	722,586±16,035	9.57	4.86	7.21
S06	635	403,225	665±9	685±10	664±11	357,728±10,341	416,059±8342	21.47	8.53	15.00
S07	423	179,208	493±40	486±15	455±8	216,138±36,366	196,519±3012	9.79	11.13	10.46
S01S	2117	4,480,292	2416 ± 57	2189 ± 46	2172 ± 34	5,303,756±129,709	4,780,221±121,065	−0.29	−0.54	−0.41
S02S	1693	2,867,366	1849±43	1773±17	1748±32	3,171,218±50,710	3,085,849±66,224	3.27	0.43	1.85
S03S	1270	1,612,900	1406±48	1351±23	1306±35	1,804,216±40,210	1,776,114±17,868	5.02	−0.66	2.18
S04S	1058	1,120,062	1138±32	1118±28	1121±31	1,206,572±24,516	1,228,381±26,908	5.16	1.99	3.58
S05S	847	716,850	917±33	900±27	958±40	769,005±20,219	848,788±13,294	6.82	1.56	4.19
S06S	635	403,225	630±20	632±33	671±32	397,326±1979	431,534±11.044	0.21	−1.76	−0.77
S07S	423	179,208	523±23	522±49	517±44	260,260±8163	267,421±19,594	4.67	0.91	2.79

Note: S01 to S07 (SXX), Specimens produced without support material; S01S to S07S (SXXS), Specimens produced with Support material; edge, cube edge dimension; A, nominal area; geometric heuristic projected area horizontal plane, GHeuH (%) $=\frac{(XDimYDim)-AreaH}{XDimYDim}\times 100$; geometric heuristic projected area vertical plane, GHeuV (%) $=\frac{(YDimZDim)-AreaV}{YDimZDim}\times 100$; geometric heuristic average, GHeuAv (%) $=\frac{1}{2}(\left(\frac{(XDimYDim)-AreaH}{XDimYDim}\right)+$($\frac{(YDimZDim)-AreaV}{YDimZDim}))\times 100$.

## Data Availability

The data presented in this study are available on request from the corresponding author.

## Abbreviations

The following abbreviations are used in this manuscript:

AM	Additive Manufacturing
mDs	micro-Details
MJ	Material Jetting
TO	Topology Optimization
ST	Sample Test
μAM	micro-Additive Manufacturing
DM	Digital Material
HQ	High Quality
HS	High Speed
UV	Ultraviolet Light
SUP705, SUP706	Support material
dpi	dots per inch
CAD	Computer-Aided Design
SXX	Specimens produced without support material
SXXS	Specimens produced with Support material
XDim	Dimension according to the xxaxis
YDim	Dimension according to the yyaxis
ZDim	Dimension according to the zzaxis
AreaH	Projected xy Area plane (Horizontal plane)
AreaV	Projected yz Area plane (Vertical plane)
*SD*	Standard Deviation
Re%	Relative error percentage
GHeuH	Geometric Heuristic (Horizontal plane projected area)
GHeuV	Geometric Heuristic (Vertical plane projected area)
